# Supplementary material for: An economic evaluation of an online computer-tailored smoking cessation intervention that includes message frame-tailoring: A randomized controlled trial
Source: PLOS Digit Health. 2022 Sep 1;1(9):e0000094. doi: 10.1371/journal.pdig.0000094 (PMC9931342; doi:10.1371/journal.pdig.0000094)
Supplement: S1 Table — (DOCX) [file pdig.0000094.s001.docx]

**Title:**

**An economic evaluation of an online computer-tailored smoking cessation intervention that includes message frame-tailoring**

**S1 Table.** Results from economic evaluation analyses based on 1000 bootstrap iterations.

| Type of analysis | Probability of highest NMB^a^, % | | | |
| --- | --- | --- | --- | --- |
|  | FCT | FT | CT | Control |
| Primary analysis |  |  |  |  |
| Abstinence | 11 | 4 | 85 | 0 |
| QoL (EQ-5D-5L) | 85 | 11 | 4 | 1 |
| QoL (ICECAP-A) | 60 | 4 | 34 | 3 |
| 1^st^ sensitivity analysis |  |  |  |  |
| Abstinence | 11 | 5 | 83 | 0 |
| QoL (EQ-5D-5L) | 85 | 10 | 4 | 1 |
| QoL (ICECAP-A) | 64 | 3 | 32 | 2 |
| 2^nd^ sensitivity analysis |  |  |  |  |
| Abstinence | 13 | 5 | 82 | 0 |
| QoL (EQ-5D-5L) | 46 | 11 | 19 | 25 |
| QoL (ICECAP-A) | 81 | 9 | 9 | 0 |

*Note*. FCT = Frame-tailoring & Content-tailoring. FT = Frame-tailoring. CT = Content-tailoring. EQ-5D-5L = the 5-level EQ-5D version by the EuroQoL group to assess health-related quality adjusted life years in economic evaluations. ICECAP-A = ICEpop CAPability measure for Adults, a broader measure for health-related quality adjusted life years in economic evaluations than the traditional EuroQoL measure. NMB = Net monetary benefit.

^a^ = at a willingness-to-pay threshold of 20,000 euro per health unit increase.
