# Supplementary material for: An economic evaluation of an online computer-tailored smoking cessation intervention that includes message frame-tailoring: A randomized controlled trial
Source: PLOS Digit Health. 2022 Sep 1;1(9):e0000094. doi: 10.1371/journal.pdig.0000094 (PMC9931342; doi:10.1371/journal.pdig.0000094)
Supplement: Attachment — Submitted filename: Response to reviewers.docx [file pdig.0000094.s002.docx]

**Response to review on manuscript “An economic evaluation of an online computer-tailored smoking cessation intervention that includes message frame-tailoring: a randomized controlled trial”**

We would like to thank the reviewer for his/her thorough review of our manuscript and the constructive, relevant remarks and suggestions. In the following, we will try to explain how we solved remarks indicated by the reviewer. The remarks of the reviewer are depicted first, followed by our responses in italics.

**Reviewer's comments:**

**Reviewer #1**:

1. I found parts of the Abstract is quite hard to follow, particularly the final paragraph of results, it took me several reads to understand. I think it would help the reader to understand this better by replacing ‘until a WTP of €2.000’ with ‘up to a WTP of €2.000’ – in the Abstract and throughout.

*We thank the reviewer for this helpful suggestion, which we have followed as can be read in the Abstract (line 40) as well as throughout the paper (page 13, line 287 and page 17, line 325).*

2. I was also not sure what the intervention entailed – the sentence ‘using a 2 (message frame-tailoring, i.e. how messages are presented: present vs absent) x 2 (content-tailoring, i.e. what content is presented: present vs. absent) design’ for example was hard to decipher. On first read through a short sentence explaining that the tailoring of the intervention is based on questions asked at baseline would also help. It wasn’t until I finished reading the manuscript that I understood this part of the Abstract.

*In order to enhance the readability and comprehensibility of the Abstract, we have adapted the description of our manipulation / the intervention in the Abstract, now reading ‘[…] using a 2 (message frame-tailoring, i.e. how messages are presented: autonomy-supportive vs controlling) x 2 (content-tailoring, i.e. what content is presented: tailored vs. generic) design. Both kinds of tailoring, content-tailoring and message frame-tailoring, were based on a set of questions asked at baseline’ (page 2, lines 32-34).*

3.Introduction: I struggled to understand what the intervention consisted of, particularly the difference between message frame-tailoring and content-tailoring. I understand that there is a word limit but would it be possible to make the intervention clearer, maybe by clarifying the examples?

*To increase the comprehensibility of the description of the intervention, we have added some description of the intervention, which is also described in detail in another manuscript about this RCT (1), reading: Thus, in the tailored study arms, participants receive tailored smoking cessation advice based on their answers to certain questions at baseline, such as an assessment of their level of addiction and their attitudes regarding quit smoking for content-tailoring and their need for autonomy for message frame-tailoring (7, 8)’ (page 3 lines 69-73). Moreover, we have now added the references of the papers which describe the development of the intervention and the results of the effectiveness analysis of message frame-tailoring, both also including a thorough description of the intervention.*

4. Methods: The first sentence of the Methods section suggests that the aim of the RCT is different to the aim of the economic evaluation – the economic evaluation seeks to establish the cost-effectiveness of the message frame-tailoring and content-tailoring compared to the other 3 arms, however the RCT aim is to establish (cost-)effectiveness of message frame-tailoring. I don’t see this is a problem with the manuscript if the aims were slightly different, but it stood out.

*We would like to clarify the reviewer’s potential confusion about the aims. That is, the aim of the RCT was twofold: First, to test the effectiveness of message frame-tailoring based on the need for autonomy, and second, to economically evaluate message frame-tailoring, in combination with content-tailoring and as stand-alone strategy, as compared to content-tailoring only and to no tailoring. One main difference between the two studies that aimed to fulfil these aims is that data used for the analyses varied. The effectiveness study (1) is based on data from T0, T1 and T2 (i.e., one-month follow-up), while this present economic evaluation relies on data assessed at T0, T1 and T3 (i.e., six-month follow-up). To clarify this, we have now adapted Figure 1 and added a timeline (page 5).*

The intervention was a bit clearer in the Methods section but with 4 arms I still struggled to conceptualize the different arms on the first reading – would it be possible to add a table or figure so the reader can refer to something visual?

*We thank the reviewer for this suggestion and added Fig 1, an illustration of study arms (page 4).*

It wasn’t clear how long the intervention lasted for – maybe a visual timeline would help make this clearer? Perhaps showing how long the intervention lasted for and then include T1, T2 and T3 and what was collected at each of these timepoints (ie quality of life etc).

For example, it is not clear here that quality of life is collected at baseline.

*As also described in our response to the comment above, we have added a more detailed Fig 2 including a timeline to clarify this (please see page 5 & 6).*

Did the baseline questionnaire inform the content-tailored messages only? The first sentence suggests this, however the next sentence described the theory behind the message frame-tailoring intervention. This just needs a bit of clarification to help the reader understand how the messages are informed.

*We thank the reviewer for this careful reading of our manuscript. Our description in the intervention section could have led to misunderstandings. Thus, we tried to clarify this section describing that the baseline questionnaire informed the content-tailored messages. In addition, participants’ need for autonomy was assessed in this baseline questionnaire, to inform the message frame tailoring. We have added this information about the tailoring process to the intervention section to prevent confusion. It now reads as follows: ‘In addition, participants’ need for autonomy was assessed in this baseline questionnaire to inform message frame-tailoring’ (page 4, lines 99 & 100).*

I may have missed it, but were ethics approval details included?

*Ethical approval details are included in the methods section of this manuscript (see page 6, lines 133-135), reading: ‘This RCT was approved by the Institutional Review Board (reference number2017-PC-7599) and is registered with the Dutch Trial Register (NL6512/NRT-6700).’ We have now added the reference numbers, as theses were still blinded.*

In terms of collecting resource use I was unsure of the timings of these – I read that from T1 the previous 6-months resource use was collected, then at 6 months too – is this 6 months post randomization or 6 months post intervention. Again a timeline would help with understanding this, and could reduce the word count – you could combine the timeline of the intervention, T1, T2, T3 with resource use.

*In order to enhance the reader’s understanding of our study design and timing of data collection, we have added the suggested timeline (as already mentioned in earlier responses to this reviewer’s comments). Please see Fig 2, page 5/6.*

*In addition, we would like to clarify that 6-month post randomization can be understood as 6-month post intervention as the intervention was received directly after randomization. The online intervention consists of an online assessment, followed by either tailored (experimental study arms) or generic (control) smoking cessation advice. Participants were able to finish the online intervention in approximately 20 minutes. Time point T1 is immediately post-intervention, which is thus on the same day as the intervention and baseline assessment (T0) took place. This T1 questionnaire immediately followed the participant’s completion of the online intervention. We have added more detail on the time points and the timeline (Fig 2) to the description of the intervention on page 5: ‘When randomized into one of the three experimental study groups, at baseline (see Fig 1), participants in the content-tailoring groups received tailored advice at several times throughout the intervention, which took participants approximately 20 minutes to complete. The flow of participants is illustrated in Fig 2. […]. Immediately post-intervention (T1), which is at the same day of the intervention, all participants answered questions about perceived message relevance, health care costs, and their quality of life’ (lines 109 – 112).*

Intervention costs – just out of interest were there any maintenance costs for the website or software?

*Indeed, there were maintenance costs, for instance, costs for hosting the intervention website, as described in the methods section on page 6, lines 133-140: ‘Intervention costs consisted of hosting costs for the website in which the tailoring software was integrated and hosting costs for the tailoring software itself. Intervention costs were the same for all three experimental study groups (i.e., FCT, FC, CT), namely 0.41 euro per participant. For the control group, costs were lower (i.e., 0.19 euro per participant) as for this group only the website hosting costs had to be considered as they received a generic advice for which no tailoring software was necessary.’ These costs were considered for main analysis as described on page 9, lines 204-206 under Data analysis approach, reading: ‘The primary and main analysis was conducted with complete cases only, i.e. only with participants who had complete data for both the baseline and the six-month follow-up measurement, and taking into account intervention costs.’ We did neither consider costs for the development of the intervention nor research-specific costs, as these were not necessary for intervention implementation.*

The healthcare costs section is clear, however I was expecting to read about which societal non-health care resources were used as these were mentioned but not detailed.

*From the societal perspective, costs were considered that were of importance for the society and outside the health care system or group of the publicly insured persons, for instance costs for medication and alternative health professionals. We adapted the description of costs considered fort his economic evaluation from the societal perspective: ‘we determined that a societal perspective would be used to also consider costs relevant for society, such as intervention costs, costs for medication and alternative health care providers, and participant costs’ (page 8, line 193 & 195).*

Were e-cigarette resource use included?

*No, we did not consider e-cigarette use as a resource, because we used and re-designed the original PAS intervention (Smit et al., 2010; Smit et al. 2012), which was developed before 2010 when e-cigarettes were not widely used among the Dutch population. We have decided to stick to the original PAS intervention due to its proven effectiveness, so we could test the effectiveness of adding message frame-tailoring in an isolated/ clean way. However, in a next redesign project, we feel e-cigarette use should certainly be considered.*

The sentence ‘QALYs were computed by taking either the average of ICECAP-A utility scores from the baseline and follow-up measurements, or the average EQ-5D-5L utility scores’ I found this sentence confusing – it suggests that QALYs are an average of either ICECAP-A or EQ-5Q-5L across the time points when these questionnaires were collected – rather than a more typical mean QALY for each arm at each time point and then the gain/loss calculated from that.

*We agree with the reviewer that the sentence about the calculation of QALYs could be misunderstood and we thank the reviewer for pointing this out. We have now rephrased the section reading as follows: ‘We used two different validated self-report measures to calculate utility scores and have conducted the cost-utility analyses with both measures separately: 1) the ICECAP-A, and 2) the Euro-QoL (EQ-5D-5L). The Dutch ICECAP-A (17) assessed […]. QALYs from the ICECAP-A measure were computed by taking the average of ICECAP-A utility scores from the baseline and follow-up measurements. QALYs assessed with the EuroQoL were assessed by taking the average the average EQ-5D-5L utility scores from the baseline and the follow-up measurement’ (page 7&8, line 169 – 183).*

Please could you add an explanation/justification of why you omitted the intervention costs in both of the sensitivity analyses?

*We have corrected the data analysis approach section, because in our initial submission we had mistakenly stated that the second sensitivity analysis was conducted without considering the intervention costs, which was wrong. The sentence now reads: ‘The second sensitivity analysis (called second sensitivity analysis hereafter) was performed including cases with missing values on the six-month follow-up measurement, with missing values being imputed using the last observation carried forward (LOCF) method and taking into account the intervention costs.’ (page 10, line 226). To clarify:*

- *In the main analysis, we analyzed complete cases and did consider intervention costs;*
- *In the first sensitivity analysis, we analyzed complete cases without intervention costs being considered;*
- *In the second sensitivity analysis, we analyzed all cases applying the LOCF method to impute missing data points, while again considering the intervention costs.*

*We have now corrected this in the data analysis approach section, and clarified this by listing the different analyses in a similar bullet point summary as above.*

Also, it would be good to see a sensitivity analysis using the Russell Standard using complete cases and including intervention costs – so it could be compared to the primary analysis.

*We thank the reviewer for this idea about the second sensitivity analysis. As stated above, we had conducted this suggested type of sensitivity analysis already, but had mistakenly omitted this information from the data analysis description in our initial submission. We apologize for this mistake, and have now corrected this.*

In the section ‘Economic evaluation approach’ if you have enough word count, it would be helpful to explain to the reader that if the NMB is 0 or above the intervention (i) would be considered cost-effective.

*We thank the reviewer for this suggestion and have added an explanation of the interpretation of the net monetary benefit to the description of the economic evaluation approach: ‘). The intervention can be considered cost-effective or has a high cost-utility, respectively, when the NMB is positive – thus, has a value of 0 or above’ (page 9, line 204 - 206).*

Results:

In your first paragraph, the last sentence says there was not a difference in baseline characteristics for demographics, chronic disease or smoking-related behaviours - were there any differences in other baseline characteristics (other than the QALYs and baseline costs that you mention in the next paragraph)? Or do all the baseline characteristics fall into these three categories?

*We thank the reviewer for this attentive question regarding our preliminary analyses. As described in our results section, there were indeed no differences in other baseline characteristics than described in the results section. We assessed age, sex, living arrangement and educational level as demographics. In addition, we checked for differences in chronic disease and smoking behaviour. We have added this supplementary information to the text (page 10&111, lines 249 & 250): ‘Comparison of participants in the different groups with regard to their demographics (i.e. age, sex, living arrangement and educational level), smoking-related behaviours and chronic conditions did not show significant differences between study groups.’*

Would it be possible to add a short section (one or two sentences) to summarise the main RCT results so the reader knows which intervention was more effective? I know that you have included the primary outcome of abstinence in Table 2, but a short summary earlier on will help set the context.

*We have added a short summary of our findings regarding descriptive findings of the outcomes smoking abstinence and quality of life (measured with the ICECAP tool and with the EuroQoL tool) preceding the section ‘economic evaluation’ to the results on page 13 (lines 274-278): ‘As shown in Table 2, smoking abstinence, i.e., the likelihood of people being abstinent from smoking for the last 7-days (at 6-months follow-up) was highest in the CT group, before and after bootstrapping. The cost-utility outcome, quality of life, was highest for people in the FCT group, when considering the ICECAP tool, or in the FT group when QoL was measured with the EuroQoL tool.’*

Were the differences in costs/QALYS adjusted for any baseline characteristics – for example baseline costs or QALYs, especially as there are imbalances in baseline costs and QALYs.

I was confused as to why the costs for the bootstrapped results were so different for each outcome?

Did you use different co-variates in a regression for each?

*We thank the reviewer for these thoughts and the attentive question regarding the differences in baseline costs and QALYs.*

*We have neither considered costs and QALYs assessed at baseline nor included any other co-variates, such as demographics, in the analyses. The differences in costs and QALYs faded and were not present anymore in the follow-up measurement, moreover, with the macros in Excel that were used for bootstrapping and main analyses, no co-variates could be added to these analyses. However, we assumed that not being able to consider these baseline differences in our cost analyses might not have influenced our results, due to the fading of the significant differences towards follow-up measurement.*

*Regarding the demographics, no significant differences were found at baseline, why these were not considered as co-variated in later analyses.*

If not then maybe running more bootstraps (5000?) would result in more similar, and potentially more robust, results.

*We agree with the reviewer that running 5000 bootstraps could lead to different results and potentially even more robust results. However, the analyses with 1000 bootstrapping iterations were conducted according to common practice for evaluation of digital interventions for their cost-effectiveness and cost-utility (Smit et al., 2013; De Ruijter et al. 2019; Stanczyk et al. 2014, Schulz et al. 2014) and we believe our results are more comparable to these others studies if we adhere to the same method.*

Discussion

I wasn’t sure what ‘A likely explanation for these findings as related to the cost-utility of the intervention, could be that…’ meant on pages 15-16, lines 304 to 305. Should this be ‘as related to the cost-effectiveness’?

*We thank the reviewer for this remark. When mentioning ‘as related to the cost-utility, we mean that the outcome considered was quality of life. In that case, the combination of message frame-tailoring and content-tailoring has led to highest gain (i.e., in terms of quality of life) for each euro spent. In contrast, when we considered smoking cessation as the outcome, we refer to ‘cost-effectiveness’. We have added this information to the phrasing of cost-utility to clarify (page 18, line 347): ‘(outcome: quality of life).’*

The first sentence in ‘Suggestions for future research’ initially suggests that for both cost-effectiveness and cost-utility FCT is favoured, however, it is only at a low WTP threshold, could the sentence be re-written so that it is clear early on in the sentence that it is only at a low WTP that FCT is favoured?

At a typical WTP of 20,000 Euros CT is favoured. This is repeated in the Conclusion, whilst technically correct it is a bit misleading.

*We have now rewritten the paragraphs in both sections as follows to reach a technically sound and potentially less misleading conclusion:*

*Suggestions for future research section: ‘Cost-utility results seemed to indicate the favourability of combining content-tailoring with message frame-tailoring under varying levels of the WTP. However, for cost-effectiveness (i.e., taking smoking abstinence as an outcome) this combination was only preferred over content-tailoring under relatively low WTP-thresholds (up to a WTP of €2000). Following, this does suggest some avenues for future research’ (page 18, lines: 362-367).*

*Conclusion section: ‘Both cost-utility analyses (i.e., taking quality of life as an outcome with two different quality of life scales) indicated the favourability of the novel combination of message frame-tailoring and content-tailoring. The integration of the two types of tailoring can thus be considered as providing good value for money. The cost-effectiveness analysis (i.e., taking smoking abstinence as an outcome) indicated the favourability of the novel combination of message frame-tailoring and content-tailoring only up to a WTP of €2000. When the WTP for each additional abstinent smoker is €2005 or higher, the addition of message frame-tailoring might not be worth the effort and content-tailoring only is preferred’ (pages 20 & 21, lines 412-422).*

In the ‘strengths’ section it says that intervention costs were included in the sensitivity analyses, however in the Methods section ‘Data analysis approach’ you state that intervention costs were excluded – this just needs amending to be consistent.

*We thank the reviewer for this attentive remark. As also described in our response to an earlier comment of this reviewer, we have now corrected the methods section accordingly. It seems that despite carefully reviewing our manuscript before submission, we have overseen this mistake. Thus, we did not correct the strengths section content wise, but added a phrase to enhance its readability: ‘Second, we used the bootstrapping method to minimize uncertainty in results and also performed two sensitivity analyses (with one sensitivity analysis with data from complete cases only without intervention costs and one sensitivity analysis with missing data imputed using the LOCF method and including intervention costs).’ (page 19, lines 390-395).*

**Minor typos:**

On page 15, line 293, there is a capital ‘I’ used for ‘in’, which should be lower case.

*Thank you, this is now corrected.*

On page 15 line 298, there should be a gap between ‘)’ and ‘effectiveness’.

*Thank you, this is now corrected.*

On page 15 line 303, the sentence starts ‘All several sensitivity analyses’, either ‘all’ or ‘several’ can be deleted here.

*Thank you, this is now corrected, i.e., we have deleted “several" from the sentence.*

Page 17, line 337, part of the sentence reads: ‘such as assessment of sweat, to gain….’, I was not sure what this meant or referred to.

*With the assessment of sweat, we refer to the measurement of emotions as often conducted in psychological experiments. Measuring for instance sweat on the skin or cortisol in the salvia could give more precise information about for instance perceived stress (due to feeling angry/not having the amount of autonomy needed in certain situations). We have clarified this, reading: ‘[…] such as assessment of sweat on the skin or cortisol in the salvia, to gain better insights in the positive and negative affect experienced by intervention participants (due to feeling angry/not having the amount of autonomy needed in certain situations)’ (page 19, lines: 378 - 381).*

Page 18, line 358, ‘e.g.’ is included where it isn’t needed*.*

*Thank you, this is now corrected.*

**Reviewer #3:**

The authors propose an economic evaluation of message frame-tailoring and content-tailoring on smoking cessation behavior.

*We thank the reviewer for taking the time and reading and reviewing our manuscript.*

It is not clear from the existing structure of the manuscript as to why this problem is important and what is the related literature and related gap.

*This manuscript describes an economic evaluation in terms of testing the cost-effectiveness as well as the cost-utility of a novel way of tailoring, message frame-tailoring, in the context of a digital smoking cessation intervention. As stated in the first sentence in the Abstract of this paper, there is a strong need to economically evaluate health behavior change interventions in general, and* ***digital*** *health behavior change interventions in particular (page 2, line 27 and following). To underline the importance of economic evaluations, we have now added a sentence including a reference to the introduction stating the importance of economic evaluations of health interventions for health policy decision making, reading: ‘Evidence of economic evaluations of behaviour change interventions is scarce, but needed to guide policy makers’ decision-making (9)’ (page 3, lines: 70 & 71). To clarify, the gap in the literature is that many health interventions are evaluated regarding their effectiveness, however, not regarding their cost-effectiveness. For policy makers and health insurance companies, however, economic evaluations are important to guide their decision making of whether an intervention will lead to higher quality of life or more favorable behavioural outcomes for the same amount of money spent, compared to an alternative intervention. Health budgets are increasingly constrained, therefore, knowing which intervention leads to most value for money is important for society.*

The paper can be structured better in terms of motivating the readers as well as discussing about terms such as economic evaluation, message framing, and content framing.

*We have now expanded our description of economic evaluation, message frame-tailoring and content-framing in the introduction, reading: ‘[…] message frame-tailoring (7) – a novel technique of tailoring, adjusting* ***how*** *information is presented to people based on their individual characteristics. That is done, for instance, by adding words that are meant to communicate decisional freedom for people with a high need for autonomy or by taking a more directive communication style for people with a lower need for autonomy – in an online smoking cessation intervention, in addition to content-tailoring, i.e., adjusting* ***what*** *information is communicated based on people’s individual situation, behaviour and beliefs. Thus, in the tailored study arms, participants received smoking cessation advice based on their answers to certain questions at baseline, such as an assessment of their level of addiction and their attitudes regarding quitting smoking as input for content-tailoring, and an assessment of their need for autonomy as input for message frame-tailoring (7, 8). Evidence of economic evaluations of online behaviour change interventions is scarce, but needed to guide policy makers’ decision-making (9). Economic evaluations measure two parameters: costs and effects, but can also determine the value for money in terms of effects. Not necessarily, the cheapest treatment or intervention option lead to the best health outcome or quality of life.’ (page 3&4, lines 62-77).*

There are multiple ambiguities about the study design and choice of constructs/variables such as how is message-framing from content-framing and why was smoking abstinence over 7-days considered as the CEA outcome and quality of life considered as CUA outcome.

*We thank the reviewer for her/his comments about the study design and outcomes. Since the main outcome in the effectiveness evaluation of this smoking cessation intervention was 7-day point prevalence abstinence from smoking, we have also considered this as the main outcome to test cost-effectiveness of the intervention. This is in line with international standards for both effectiveness evaluation (Jackson et al., 2014) and economic evaluations (Drummond et al., 2015).* *Quality of life was considered the outcome for the CUA, as this is as well general practice in cost-utility analyses (Drummond et al. 2015). Moreover, quality of life, the outcome of the cost-utility analysis was measured with 2 different and validated quality of life scales – the EuroQoL scale (Versteegh et al., 2016) and the ICECAP scale (Al Janabi, Flynn & Coast, 2012; Engel et al., 2017; Flynn et al., 2015) - both measuring different aspects of quality of life. This gave more insight into participants “real” quality of life.*

*With regards to the reviewer’s comment regarding the study design and choice of constructs/variables “such as how is message-framing from content-framing” we addressed this point in our response above by adding an elaboration of our description of message frame tailoring and content-tailoring to the introduction (see response above or manuscript page 3 lines 62 and following). We hope that we have responded to your comment well. In case our response seems not to be satisfying, we would kindly ask the reviewer elaborate on his/her comment, so we would be better able to respond to the reviewer’s request.*

Finally, though the title reads computer-tailored, the digital health aspect is either missing or not clear in this study. That is, the study could have been conducted using messages and contents printed on paper-surveys as medium.

*We thank the reviewer for this observation. This intervention is a digital intervention since the message frame-tailoring, as well as the content-tailoring, was an automated – computer-tailored – process, which could not have been easily (or in a handy way for respondents) be printed out. The Web-based smoking cessation was exclusively accessible via the project website and was suitable for computers, laptops, as well as for mobile phones and tablets. Tailoring was done at several moment throughout the intervention and based on an assessment concerning participants’ smoking behavior, attitude, self-efficacy, social influence, action and coping planning, as well as their intention to quit smoking or to remain a nonsmoker. As well as the individual need for autonomy (message frame-tailoring). Thus, the tailored advice was different for each individual/participant and could not have been printed out on paper in an economical way. The choice for creating and testing an online intervention is due to the specifically promising features of the online environment, with a potentially great reach, an “easy to access” medium and thus a potentially large impact on public health (Smit, Linn, van Weert, 2015; Taylor et al., 2017).*

I would recommend the paper to be rejected in its current form as it does not appear to align with mission and scope of the journal, does not have significant results, and lacks clarity in current form.

*We thank the reviewer for sharing his/her thoughts on the results of our study and the quality of our paper. However, we argue that also non-significant results, which concerns rather the results of the effectiveness study (published elsewhere: Altendorf et al., 2020) and not the results of this economic evaluation, are worth publishing. Non-significant findings are just findings – in the same way significant findings are. Moreover, not publishing non-significant findings leads to a publication bias and a waste of (public) money. However, we are not sure, how the reviewer comes to the conclusion that our economic evaluation shows non-significant results, besides the reported non-significant differences in costs at follow up (page 10, line 245), because our economic evaluation in fact showed differences between intervention arms in terms of cost-effectiveness and cost-utility, which can be found in Figure 3 (results section).*

*As regards to the clarity of our manuscript, we have done our best to improve this based on the comments from this and the other reviewer, and we hope – and are confident – that we have now taken away this concern.*

**References**

Al-Janabi H, Flynn T, Coast J (2012). Development of a self-report measure of capability wellbeing for adults: the ICECAP-A. Quality of Life Research, 21(1), 167-176.

Altendorf M, Hoving C, Van Weert JC, Smit ES. Effectiveness of Message Frame-Tailoring in a Web-Based Smoking Cessation Program: Randomized Controlled Trial. J Med Internet Res 2020;22(4):e17251. doi: 10.2196/17251

De Ruijter, D, Hoving, C, Evers, S, Hudales, R, de Vries, H, & Smit, ES (2019). An economic evaluation of a computer-tailored e-learning program to promote smoking cessation counseling guideline adherence among practice nurses. Patient Education and Counseling, 102(10), 1802-1811.

Drummond, M, Sculper, MJ, Claxton, K, Stoddart, GL, Torrance GW (2015). Methods for the economic evaluation of health care programmes. Fourth Edition. Oxford University Press: UK.

Flynn, TN., Huynh, E, Peters, TJ, Al‐Janabi, H, Clemens, S, Moody, A, & Coast, J (2015). Scoring the ICECAP‐A capability instrument. Estimation of a UK general population tariff. Health economics, 24(3), 258-269.

Engel, L., Mortimer, D., Bryan, S., Lear, S. A., & Whitehurst, D. G. (2017). An investigation of the overlap between the ICECAP-A and five preference-based health-related quality of life instruments. PharmacoEconomics, 35(7), 741-753.

Jackson, D, White, IR, Manson, D, Sutton, S (2014). A general method for handling missing binary data in randomized controlled trials. Addiction, 109, 1986 – 1993.

Schulz DN, Smit ES, Stanczyk NE, Kremers SP, de Vries H, Evers SM. Economic evaluation of a web-based tailored lifestyle intervention for adults: findings regarding cost-effectiveness and cost-utility from a randomized controlled trial. J Med Internet Res. 2014 Mar 20;16(3):e91. doi: 10.2196/jmir.3159. PMID: 24650860; PMCID: PMC3978559.

Smit, ES, De Vries, H, & Hoving, C (2010). The PAS study: A randomized controlled trial evaluating the effectiveness of a web-based multiple tailored smoking cessation programme and tailored counselling by practice nurses. Contemporary Clinical Trials, 31(3), 251-258. <http://doi.org/10.1016/j.cct.2010.03.001>.

Smit, ES, de Vries, H, & Hoving, C (2012). Effectiveness of a Web-based multiple tailored smoking cessation program: a randomized controlled trial among Dutch adult smokers. Journal of medical Internet research, 14(3), e82.

Smit, ES, Evers, S M, de Vries, H, & Hoving, C (2013). Cost-effectiveness and cost-utility of Internet-based computer tailoring for smoking cessation. Journal of medical Internet research, 15(3), e57.

Smit, E. S., Linn, A. J., & van Weert, J. C. (2015). Taking online computer-tailoring forward: The potential of tailoring the message frame and delivery mode of online health behaviour change interventions. European Health Psychologist, 17(1), 25-31.

Stanczyk NE, Smit ES, Schulz DN, de Vries H, Bolman C, Muris JW, Evers SM. An economic evaluation of a video- and text-based computer-tailored intervention for smoking cessation: a cost-effectiveness and cost-utility analysis of a randomized controlled trial. PLoS One. 2014 Oct 13;9(10):e110117. doi: 10.1371/journal.pone.0110117. PMID: 25310007; PMCID: PMC4195710.

Taylor G, Dalili M, Semwal M, Civljak M, Sheikh A, Car J. Internet-based interventions for smoking cessation. Cochrane Database Syst Rev 2017 Sep 04;9:CD007078

Versteegh, MM, Vermeulen, KM., Evers, S, de Wit, GA, Prenger, R, & Stolk, EA (2016). Dutch tariff for the five-level version of EQ-5D. Value in health, 19(4), 343-352.
